# Estimation of Salt Intake in Normotensive and Hypertensive Children: The Role of Body Weight

**DOI:** 10.3390/nu15030736

**Published:** 2023-02-01

**Authors:** Martina Kos, Tihana Nađ, Lorena Stanojević, Matea Lukić, Ana Stupin, Ines Drenjančević, Silvija Pušeljić, Erna Davidović Cvetko, Zrinka Mihaljević, Dijana Dumančić, Ivana Jukić

**Affiliations:** 1 Clinic of Pediatrics, University Hospital Osijek, J. Huttlera 4, HR-31000 Osijek, Croatia; 2 Department of Pediatrics, Faculty of Medicine Osijek, University Josip Juraj Strossmayer Osijek, J. Huttlera 4, HR-31000 Osijek, Croatia; 3 Faculty of Medicine Osijek, University Josip Juraj Strossmayer Osijek, J. Huttlera 4, HR-31000 Osijek, Croatia; 4 Institute and Department of Physiology and Immunology, Faculty of Medicine Osijek, University Josip Juraj Strossmayer Osijek, J. Huttlera 4, HR-31000 Osijek, Croatia; 5 Scientific Centre of Excellence for Personalized Health Care, University of Osijek, Trg Sv. Trojstva 3, HR-31000 Osijek, Croatia; 6 Lavoslav Ružička College of Applied Sciences of Vukovar, Županijska 50, HR-32000 Vukovar, Croatia; 7 Department of Diagnostic and Interventional Radiology, University Hospital Osijek, J. Huttlera 4, HR-31000 Osijek, Croatia; 8 Department of Radiology, Faculty of Medicine Osijek, University Josip Juraj Strossmayer Osijek, J. Huttlera 4, HR-31000 Osijek, Croatia

**Keywords:** children, essential arterial hypertension, 24-h urine, dietary sodium, food frequency questionnaire

## Abstract

Objective: The connection between increased dietary salt intake and arterial hypertension has been recognized for a long time, even in children. This study aimed to investigate salt consumption in normotensive and hypertensive children and evaluate their dietary habits. Materials and Methods: A total of fifty participants were included in this cross-sectional study: twenty-five normotensive children and 25 children of both sexes with essential arterial hypertension from 12–17 years old. Subjects’ body mass index, waist-to-hip ratio, body composition and arterial blood pressure were measured, and their daily salt intake was calculated from 24-h urine samples. Using the food frequency questionnaire (FFQ), the data on the average daily total energy and food intakes were collected and analyzed using a suitable program. Results: Estimated daily salt intake was significantly higher in hypertensive compared to normotensive children, and this is positively associated with blood pressure and body mass index (BMI). Hypertensive children had significantly higher BMIs, which also positively correlated with blood pressure. Consistently, resting metabolic rate (kcal) is higher in hypertensive children compared to normotensive, and this is also associated with blood pressure. Reported energy intake is also enlarged in hypertensive compared to normotensive children and for both groups, levels are significantly higher than the recommended values. Conclusions: Our study results confirm the relationship between daily salt consumption, blood pressure and body weight. Sodium consumption related to blood pressure and body weight among children. Cardiovascular disease prevention should start in early childhood by reducing salt intake and preventing overweight/obesity since these are two of the most important modifiable risk factors for hypertension.

## 1. Introduction

Increased salt intake is globally accepted as the main determinant for elevated blood pressure (BP) and hypertension [1,2] and it is associated with various cardiovascular disease (CVD) outcomes [3,4]. Some studies have demonstrated an association between excessive salt intake and elevated BP and the prevalence of pediatric hypertension [5,6,7]. This is important since it is known that essential hypertension in childhood is associated with hypertension in adulthood [8]. The World Health Organization (WHO) recommends salt consumption of less than 5 g per day (approximately 2 g sodium) to decrease blood pressure and reduce the risk of CVD [9]. The average salt consumption in the adult population in Croatia is 11.6 g [10], which is consistent with the estimation that individuals consume between 6 and 12 g of salt daily worldwide [11,12], thus exceeding the WHO-recommended levels. However, data on salt intake in children are limited and due to unhealthy dietary habits in the pediatric population, it is suggested that salt intake exceeds the recommended amount [13]. Furthermore, the target intake should be adjusted according to the energy requirements of children [5,9].

The “gold standard“ in the evaluation of dietary sodium (Na) intake is the determination of excreted sodium in 24-h urine [14]. With the exception of increased daily salt intake, an inadequate intake of potassium (K) has harmful effects on arterial BP [15,16]. The urinary ratio of sodium-to-potassium (Na-to-K) is considered to be a more precise predictor of CVD risk than Na or K intake alone [17]. The target Na-to-K ratio still remains a matter of debate but a ratio of less than 1.0 has been suggested as the best balance of Na and K intake for the prevention of CVD [18]. Since dietary habits are acquired during childhood [19], including the tendency toward eating salty food [20], education about the importance of a low salt diet and an adequate intake of potassium during childhood is imperative. Appropriate daily consumption of sodium and potassium represents a lifestyle recommended for the prevention and treatment of hypertension and CVD [21]. It has been shown that excess dietary sodium consumption is associated with elevated BP [7,22] and lower sodium intake is associated with reduced BP in children [23]. Elevated potassium consumption is also related to BP and has a beneficial effect on BP in adults, but in children, evidence of the relationship between salt and potassium consumption and BP is miscellaneous. A Greek cross-sectional study involving 606 children has demonstrated the positive connection between potassium intake and systolic BP (SBP), whereas no connection was demonstrated between sodium intake and SBP [24]. A longitudinal study of 233 children with annual 24-h urine samples measured over a follow-up period of 7 years did not show a connection between sodium excretion and a change in BP, whereas higher potassium intake lowers mean systolic BP [25]. On the other hand, a meta-analysis of randomized controlled trials and cohort studies found a non-significant connection between higher potassium intake and reduced systolic blood pressure [26]. Since the data are very limited and inconsistent, further studies are needed to highlight dietary habits so that mitigation measures toward the prevention of hypertension in childhood and future cardiovascular (CV) events could be undertaken.

In addition to the high prevalence of hypertension in children [27], obesity has become a significant global public health challenge. Even among children there is an increased incidence of overweight/obesity and its associated metabolic complications [28,29]. It is well known that blood pressure values are shifted towards higher levels as body weight enhances [30]. Obesity is related to the high incidence of CV risk factors, including hypertension, and in overweight adolescents, a positive correlation between body mass index (BMI) and BP [31] is shown. Importantly, studies on sodium consumption and its relation to body weight have demonstrated that a high sodium intake is connected with overweight and obesity status in children and young people [32,33]. Since blood pressure trails from childhood to adulthood, it is suggested that reduced salt consumption early during childhood could lower blood pressure and prevent the onset of hypertension later in life.

Available evidence from studies conducted in children on salt consumption and its connection with body weight and blood pressure are limited. Thus, the present study aimed to evaluate daily salt intake and its relationship with body weight and blood pressure in healthy normotensive children and in children with essential arterial hypertension relating to their dietary habits, estimated by a food frequency questionnaire (FFQ).

## 2. Methods

### 2.1. Study Participants

This study involved fifty subjects divided into 2 groups: healthy normotensive children (N = 25) allocated to the NT (normotensive) group and children with confirmed essential arterial hypertension (N = 25) allocated to the HT (hypertensive) group; subjects included both sexes, aged 12–17 years. The participants were recruited during their regular visits to a pediatrician at the Clinic of Pediatrics, University Hospital Osijek, Osijek, Croatia. The most common reason for normotensive children to visit a pediatrician was tension headaches, while hypertensive children were recruited during regular follow-up visits to a pediatric nephrologist. The participating pediatricians were thoroughly informed about the study and were asked to recruit subjects according to the given inclusion/exclusion criteria among their parents/guardian. Their parents/guardian were informed of the protocols and procedures this study involved in detail and they signed informed consent.

Hypertension was defined as a 24-h systolic and/or diastolic blood pressure (DBP) equal to or greater than the 95th percentile for age, height and sex measured on three or more separate occasions, or a blood pressure exceeding 130/80 mmHg [34].

Children with secondary causes of hypertension (renal, renovascular, endocrinological, cardiological or neurological), white coat hypertension and masked hypertension were excluded from the study.

The protocols and procedures used in this study were in accordance with the latest revision of the Declaration of Helsinki and approved by the Ethical Committee of the University Hospital Osijek, Osijek, Croatia (R1/6414/2021) and the Ethical Committee of the Faculty of Medicine Josip Juraj Strossmayer University of Osijek, Osijek, Croatia (Cl: 602-04/21-08/07; No: 2158-61-07-21-06). The present study is a part of the registered clinical trial database at ClinicalTrials.gov, investigating the effect of juvenile arterial hypertension on vascular function (ID NCT05109013 Juvenile Essential Arterial Hypertension and Vascular Function).

### 2.2. Anthropometric and Arterial Blood Pressure Measurement

Using a scale (Personal Scale, Radwag, Radom, Poland), all subjects were weighed (kg) without shoes and with light clothes, and their height (m) was measured. Using the formula weight/(height)^2^ (kg/m^2^) [35], the body mass index, BMI, was calculated. Futhermore, all subjects were measured for waist and hip circumference (cm) in order to calculate waist-to-hip ratios (WHR). Arterial blood pressure and heart rate (HR) were measured using an automated oscillometric sphygmomanometer (OMRON M3, OMRON Healthcare Inc., Osaka, Japan) after a 15-min rest in a seated position. The final BP and HR was an average of three consecutive measurements. Using the formula, MAP = [SBP + 2 (DBP)]/3 [36], the mean arterial pressure (MAP) was calculated. Children with arterial hypertension had their blood pressure measured using a 24-h continuous blood pressure monitor (GE Tonoport V, GE HealthCare, Chicago, IL, USA).

### 2.3. Body Composition and Body Fluid Status Measurements

For the measurement of body composition and body fluid status estimation, a four-terminal portable impedance analyzer (Maltron Bioscan 920-II, Maltron International Ltd.; Rayleigh, Essex, UK) was used. It is a non-invasive and rapid method that uses empirically derived formulas (the original manufacturer’s software) to calculate resting metabolic rate (RMR kcal), fat-free mass% (FFM%), fat mass% (Fat%), total body water (TBW%), extracellular water (ECW%), intracellular water (ICW%), plasma fluid (PF), interstitial fluid (IF) and body density (kg/L).

### 2.4. 24-h Urine Sample Analysis

Urine was collected during the 24-h period according to the given instructions outlined by the WHO [37] in a standard, sterilized urine collection bottle. Measured parameters included 24-h urine sodium, potassium, creatinine coefficients and protein concentrations. Sodium and potassium molar excretion in 24-h urine was used for the estimation of daily sodium and potassium consumption in milligrams (mg) using appropriate formulas [1 mmol = 22.99 mg of sodium or 39.10 mg of potassium], and daily salt intake was estimated based on 24-h urinary sodium excretion [1 g salt (NaCl) = 393.4 mg Na = 17.1 mmol Na]; salt intake can then be calculated: salt (g/day) = 24-h urinary sodium (mmol/24-h)/17.1) [38]. The sodium to potassium ratio was also calculated. The 24-h urine samples were analyzed at the Clinical Institute of Laboratory Diagnostics, University Hospital Osijek, Osijek, Croatia.

### 2.5. Dietary Assessment

Data on the average daily total energy and food intakes were obtained with a validated EPIC-Norfolk food frequency questionnaire. The FFQ provides data on the frequency of the consumption of different foods as well as their quantity. For each subject, the FFQ was used to estimate the average intake of foods during the previous year. It consisted of a list of foods and standard serving sizes for each. The list of food contains 10 categories: (1) meat and fish, (2) bread and savory biscuits, (3) cereals, (4) potatoes, rice and pasta, (5) dairy products and fats, (6) sweets and snacks, (7) soups, sauces, and spreads, (8) drinks, (9) fruits and (10) vegetables. The participants were asked to report the frequency of consumption of a given serving of each food item during the previous year on a daily, weekly or monthly basis. In the second part of the questionnaire, there are additional detailed questions related to the use of certain foods and supplements used during the last year. The coded data then were processed using appropriate software, i.e., the FFQ EPIC Tool for Analysis (FETA) [39]. The results included average nutrient values for the last year in their recommended units.

### 2.6. Statistical Analysis

Results were reported as medians and min to max ranges. The Mann–Whitney U test was used for data analysis, and an analysis of covariance (ANCOVA) adjusted for age and sex was performed. Spearman’s correlation test was used for analysis of the correlations between variables. *p* < 0.05 was considered statistically significant. Statistical analysis was performed using SigmaPlot, version 11.2 (Systat Software, Inc., Chicago, IL, USA).

## 3. Results

### 3.1. Characteristics of Study Participants

Fifty children were included in the present study and divided into two groups according to the presence/absence of essential arterial hypertension: the NT group—normotensive children (N = 25), 13 girls and 12 boys; and the HT group—hypertensive children (N = 25), 10 girls and 15 boys. Their sexual maturity was determined according to Tanner Staging and there was no difference between NT (4 [3,4]) and HT (4 [3,4]) groups.

Participants’ anthropometric and hemodynamic parameters are presented in Table 1. As expected, systolic BP, diastolic BP and mean BP were significantly higher in the HT compared to the NT group, while heart rate was similar in both groups. Children with essential arterial hypertension had significantly higher BMIs and WHRs than normotensive children. Median BMI z-scores in the NT group were 0.7 [–2.88–2.69] and 1.93 in the HT group [–2.27–2.84].

Systolic, diastolic and mean BP significantly positively correlated with BMI (SBP r = 0.586, *p* < 0.001; DBP r = 0.356, *p* = 0.016; MAP r = 0.488, *p* < 0.001) and WHR (SBP r = 0.321, *p* < 0.05; DBP r = 0.286, *p* = 0.044; MAP r = 0.351, *p* < 0.05).

### 3.2. 24-h Urine Assessment

Results of the 24-h urine analysis are presented in Table 2. Creatinine coefficient and protein excretion values were similar in the NT and HT groups. 24-h urine sodium and potassium excretion, as well as calculated daily salt consumption were significantly higher in the HT group compared to the NT group. According to 24-h urine analysis, the median molar Na-to-K ratio was higher in the NT group compared to the HT group.

Calculated daily salt intake significantly positively correlated with SBP (r = 0.517, *p* < 0.001), DBP (r = 0.441, *p* < 0.001), MAP (r = 0.524, *p* < 0.001) and BMI (r = 0.443, *p* = 0.003).

### 3.3. Body Composition and Body Fluid Status

The body composition and body fluid status of subjects are presented in Table 3. Resting metabolic rate was significantly higher in the HT (1935 [1456–2529] kcal) compared to the NT group (1633 [1425–2605] kcal). Fat mass (%), plasma fluid (L) and interstitial fluid (L) were significantly higher in the HT compared to the NT group, while fat-free mass (%), total body water (%) and body density (kg/L) were significantly higher in the NT compared to the HT group. There were no significant differences in extracellular and intracellular water (%) between the groups.

Except with BMI (r = 0.538, *p* < 0.001), RMR significantly positively correlated with systolic BP (r = 0.567, *p* < 0.001) and mean BP (r = 0.478, *p* = 0.001). Systolic and mean BP significantly positively correlated with fat mass (SBP r = 0.381, *p* = 0.017; MAP r = 0.324, *p* = 0.047) and negatively correlated with fat-free mass (SBP r = −0.369, *p* = 0.017; MAP r = −0.364, *p* = 0.023). There was no significant correlation between diastolic BP and measured body composition and body fluid status parameters.

### 3.4. Food Frequency Questionnaire (FFQ)

Daily energy and selected nutrient intake in normotensive and hypertensive children assessed by the EPIC-Norfolk food frequency questionnaire are presented in Table 4. Reported energy intake was significantly higher in the HT group (5965 [914–7473] kcal) compared to the NT group (2445 [1072–3625] kcal). Participants from both groups consumed less than the advised range of mean energy intake from carbohydrates (45–65% is recommended) and protein (10–30% is recommended), while energy intake from fat was significantly higher than the recommended 25–35%. Furthermore, the distribution (%) of the basic sources of energy—carbohydrates, proteins and fats—is similar in both groups. Individuals from the HT group have a significantly higher daily intake of cereals and cereal products, fats and oils, meat and meat products, soups and sauces, sugars and vegetables than individuals from the NT group. The daily intake of eggs and egg dishes, fish and fish products, fruits, milk and milk products are similar in both groups.

## 4. Discussion

Worldwide, there is little evidence on sodium and potassium consumption in children and adolescents, and the existing data are mostly from developed countries [40]. In Croatia, to our best knowledge, this is the first study to estimate sodium/salt intake and its connection with body weight and blood pressure in children using the 24-h urinary excretion approach, while also including children with essential arterial hypertension.

In this study, the estimation of sodium status in children and adolescents plainly demonstrated that the average daily sodium intake is higher than the recommended values [9]. Using a 24-h urinary sodium excretion, the estimated daily sodium intake was 2736 [2062–3770] mg/day for normotensive children. This is consistent with previously published data that indicate a largely average daily sodium intake among children in Europe between 2400 and 3000 mg/day [41,42,43,44]. In children with hypertension, the estimated daily sodium intake was 4195 [1559–5783] mg/day, which is significantly higher than in normotensive children. We should be concerned about this data since it is well known that high sodium intake is a risk factor for elevated blood pressure/hypertension in adults [45,46] and additionally, a positive association between sodium consumption and blood pressure in childhood has been found [6,22,47]. In the present study, the estimated daily salt intake was 7.09 [5.25–9.59] g/day for normotensive children and 10.7 [4.0–14.7] g/day for children with essential arterial hypertension, demonstrating that both groups of children consume more salt than the World Health Organization (WHO) recommendation [9]. Furthermore, the estimated daily salt intake significantly positively correlated with systolic, diastolic and mean arterial BP, which is consistent with previously published results from experimental and observational studies [6,41,48,49]. Therefore, reducing the salt intake in children’s diets could be an important step in the prevention of hypertension.

Excessive salt intake is a global problem present all over the world, including Croatia [10]. A study on children’s dietary habits showed that school children from Eastern Croatia have some bad dietary habits, including eating bakery products every day (in 51.6% children) and thus consuming additional salts [50]. Although it has been demonstrated that sodium consumption in children is 100–250% higher than recommendations [51,52,53,54,55], it should be noted that recommendations for salt consumption in children are based on an extrapolation of results obtained from adults. Moreover, there are no differences between the recommendations for healthy children and obese and/or hypertensive children [56]. In addition to the association with hypertension, it has been demonstrated that daily salt consumption in children is associated with the prevalence of obesity [41]. There is a direct connection between overweight/obesity and essential hypertension, which is in accordance with our results. In the present study, the assessed daily salt consumption significantly positively correlated with BMI, which was significantly greater in hypertensive children compared to normotensive children. Regarding BMI z-scores, the participants were classified as underweight/normal weight if their z-scores were between −2 and +0.99. Those with BMI z-scores between 1 to 1.99 were considered overweight, those from 2 to 2.99 obese, and those from ≥3 very obese children [57]. Our study results indicate that hypertensive children are mostly overweight or obese, while most normotensive children have a normal body weight. This observation is in accordance with earlier studies [58,59]. Furthermore, studies demonstrated that body composition is notably associated with various health outcomes [60,61]. In the present study we confirmed the differences between the body composition parameters of hypertensive children and healthy normotensive children. Hypertensive children had a significantly higher percentage of fat mass compared to normotensive children, whereas the percentage of fat-free mass was significantly lower compared to normotensive children. Furthermore, SBP and MAP significantly positively correlated with fat mass, which is consistent with other studies [60,62,63]. However, while some reported on the association between diastolic BP and fat tissue content [62], this was not shown in our study due to a preponderance of isolated systolic hypertension. Our results indicate that body composition plays a significant role in the etiology of the disease and is also possibly an additional parameter to monitor treatment improvement.

Among children, the admissible macronutrient distribution range for carbohydrates, proteins and fats is 45–65%, 10–30% and 25–35% [64]. Our study reported that the intake of carbohydrates, proteins and fats (expressed as a percentage) was similar in normotensive and hypertensive children. Furthermore, both groups consumed proteins within the recommended interval (NT 17%, HT 14%), while carbohydrate intake was slightly lower than the recommended reference (NT 40%, HT 42%). On the other hand, normotensive and hypertensive children consumed fats above the upper limit and our study reports an increased mean caloric intake compared to recommendations, for both groups. It is interesting, but also alarming, to compare resting metabolic rate with reported energy intake. Namely, reported energy intake for the NT group was 2445 [1072–3625] kcal, while RMR was 1633 [1425–2605] kcal. For the HT group, reported energy intake was 5965 [914–7473] kcal, while RMR was 1935 [1456–2529] kcal, which is very concerning. RMR represents a significant component of total energy expenditure that contributes 65–75% of the total daily energy demands [65]. Hypertension is connected with an enlarged resting metabolic rate (RMR) [66,67,68], which is confirmed in the present study. Taking this into account, as well as the BMI values, we can conclude that hypertensive children consume significantly more calories than they should, which certainly has a significant impact on their health condition.

Recent studies indicate that the Na-to-K ratio is more reliable than sodium or potassium alone in the assessment of blood pressure outcomes and hypertension [17,69]. However, it remains to be investigated whether this is true in normotensive adults and children [70] since the existing reports are inconsistent. A cross-sectional study of healthy schoolchildren from northern Greece aged 7–15 years reported a positive association between systolic blood pressure and potassium intake [24]. On the other hand, a longitudinal study on children in which 24-h urine was collected during a follow-up period of seven years reported that systolic BP was lower when potassium intake was higher [25]. Trying to clarify at least part of this question, we also assessed 24-h urinary potassium excretion and estimated daily potassium consumption. The mean potassium excretion in normotensive children was 30.9 [22.8–37.9] mmol/dU and 63.8 [23.5–92.3] for hypertensive children and therefore, the estimated daily potassium intake was significantly higher in hypertensive children (2497 [919–3609] mg) compared with normotensive children (1208 [891–1482] mg). As a result of the increased sodium, but also increased potassium intake, in hypertensive children, the median molar 24-h urinary Na–K ratio was lower in hypertensive (3 [1.3–6.6]) compared with normotensive (4 [2.61–5.4]) children. Interestingly, other countries in Europe did not achieve the recommended Na-to-K ratio; the average Na-to-K ratio in Spanish and French children (2–14 years) were 3.6 and 1.64, respectively [71,72]. On the other hand, it was reported that the average of Na-to-K ratio in American children is 1.03 [22], and a low consumption of sodium and potassium was reported for children in rural South Africa [73]. Dickinson and colleagues [74] concluded that the evidence on the beneficial effect of potassium supplementation on BP are not convincing and high-quality controlled trials are needed to examine the association between potassium supplementation and blood pressure lowering and better health outcomes. However, considering the dietary habits of our participants, our study results are not so unexpected. Although the reported energy intake exceeded the recommended values [75] for both groups, results of the daily dietary intake assessed by FFQ reported that hypertensive children consumed significantly more calories and have generally ate more than normotensive children. In addition to eating a lot of sodium, they also had a high intake of potassium.

Although over the last few years there has been increased interest in childhood hypertension, it is still insufficiently recognized as an important and detrimental health condition. The prevalence of primary hypertension in adolescents (especially near their twenties) is greatly similar to that in adults, which in Croatia is 37.5% [10]. The onset of hypertension should be addressed at a much earlier age than 18 years. On the other hand, we are witnessing a global obesity pandemic and a high prevalence of obesity among Croatian children [76]. The childhood obesity epidemic has led to an increased prevalence of arterial hypertension and its consequences on children and adolescents [77]. Importantly, in children, the increased salt consumption by 1 g is connected with a 0.4 mm Hg increase in systolic BP [78]. Our study, and others [79,80], emphasize the excessive salt intake in children, indicating the need for significant intake restriction that should reduce the cardiovascular risk. Taken together, cardiovascular disease prevention should be aimed at early childhood, and it should include measures to prevent overweight/obesity and lower salt intake in childhood. Therefore, establishing healthy eating habits in childhood are suggested to be an important step in the prevention of arterial hypertension and its associated consequences.

The present study is very informative, and its main strength relates to its technical approach to the evaluation of sodium and potassium consumption in children using an objective assessment, 24-h urinary excretion. Moreover, these obtained data are extremely valuable since children are a vulnerable population and parents/guardians are often hesitant about participating in research. However, the main limitation of the present study is its small sample size and further studies including a larger number of participants are necessary to draw consistent conclusions regarding the relationship between children’s nutrition/salt consumption and blood pressure values.

## 5. Conclusions

In conclusion, although very challenging, this is the first study to estimate daily salt consumption from a 24-h urine sample in children in Croatia. The present study has demonstrated that children in Croatia, both normotensive and hypertensive, consume more salt than the recommended values. It is certainly important to emphasize that hypertensive children, in addition to excessive salt intake, generally consume too much food and have an increased body weight compared to normotensive children. Moreover, reported energy intake is above the upper limits even for normotensive children, and especially for hypertensive children, indicating the presence of overeating in Croatian children. Considering the detrimental consequences of obesity and hypertension, it is of the utmost importance to fortify individual and public health efforts to increase awareness of health hazards and provide education on healthy lifestyle and dietary habits to prevent hypertension and obesity starting in early childhood.

## Figures and Tables

**Table 1:** Anthropometric and hemodynamic parameters of the subjects.

Parameter	NT Group	HT Group
N (F/M)	25 (13/12)	25 (10/15)
Age (years)	16 [12–17]	16 [13–17]
Body mass index (kg/m^2^)	21.48 [16.0–40.46]	28.84 [15.24–42.87] *
Waist-to-hip ratio	0.79 [0.69–1.12]	0.84 [0.71–0.95] *
Systolic blood pressure (mmHg)	111 [79–125]	130 [125–156] *
Diastolic blood pressure (mmHg)	72 [56–89]	80 [62–100] *
Mean arterial pressure (mmHg)	85 [75–98]	97 [83–113] *
Heart rate (beats per min)	87 [63–107]	87 [55–110]

Data are presented as medians and min-max ranges; * *p* < 0.05. NT—normotensive; HT—hypertensive; N—number of participants; F—female; M—male.

**Table 2:** 24-h Urine Biochemical Parameters.

Parameter	NT Group	HT Group
Creatinine Coefficient (µmol/24 h/kg)	178 [123–196]	160 [113–266]
Proteins (mg/dU)	92 [63–134]	95 [30–209]
Sodium (mmol/dU)	121.2 [89.7–164]	183 [67.8–251.5] *
Potassium (mmol/dU)	30.9 [22.8–37.9]	63.8 [23.5–92.3] *
Estimated daily sodium intake (mg)	2736 [2062–3770]	4195 [1559–5783] *
Estimated daily potassium intake (mg)	1208 [891–1482]	2497 [919–3609] *
Estimated daily salt intake (g/day)	7.09 [5.25–9.59]	10.7 [4.0–14.7] *
Sodium-to-potassium ratio (molar ratio)	4 [2.61–5.4]	3 [1.3–6.6] *

Data are presented as medians and min-max ranges; * *p* < 0.05. NT—normotensive; HT—hypertensive; N—number of participants.

**Table 3:** Body composition and body fluid status of the subjects.

Parameter	NT Group	HT Group
RMR (kcal)	1633 [1425–2605]	1935 [1456–2529] *
Fat-free mass (%)	82.53 [59.68–94.64]	72.87 [48.80–94.15] *
Fat mass (%)	17.47 [5.36–40.33]	26.48 [5.85–42.29] *
Total body water (%)	63.05 [44.78–80.00]	55.58 [40.39–75.72] *
Extracellular water (%)	42.82 [40.49–52.38]	44.72 [39.84–52.22]
Intracellular water (%)	57.17 [47.61–59.50]	55.27 [47.77–60.15]
Plasma fluid (L)	3.34 [2.48–5.86]	3.77 [2.88–6.07] *
Interstitial fluid (L)	11.69 [8.68–20.51]	13.19 [10.08–21.24] *
Body density (kg/L)	1.06 [0.99–1.09]	1.03 [0.99–1.09] *

Data are presented as medians and min-max ranges; * *p* < 0.05. NT—normotensive; HT—hypertensive; RMR—resting metabolic rate.

**Table 4:** Daily dietary intake assessed by EPIC-Norfolk Food Frequency Questionnaire (FFQ).

	NT Group	HT Group
Total energy intake (kcal/day)	2445 [1072–3625]	5965 [914–7473] *
Protein (% of total energy/day)	17%	14%
Carbohydrates (% of total energy/day)	40%	42%
Fats (% of total energy/day)	43%	44%
Cereals and cereal products (g/day)	188 [98–459]	810 [129–1115] *
Eggs and egg dishes (g/day)	32 [18–61]	22 [0–61]
Fats and oils (g/day)	29 [12–40]	86 [4–116] *
Fish and fish products (g/day)	41 [0–111]	72 [19–96]
Fruit (g/day)	143 [83–308]	97 [44–517]
Meat and meat products (g/day)	223 [94–457]	514 [52–786] *
Milk and milk products (g/day)	213 [11–821]	213 [25–756]
Soups and sauces (g/day)	132 [59–137]	189 [41–246] *
Sugars (g/day)	94 [13–123]	145 [17–197] *
Vegetables (g/day)	187 [55–221]	289 [81–464] *

Data are presented as medians and min-max ranges; * *p* < 0.05. NT—normotensive; HT—hypertensive.

## Data Availability

The data presented in this study are available upon request from the corresponding author.
